# CNS distribution and preclinical activity of the PI3K/AKT inhibitors inavolisib and ipatasertib in pediatric-type diffuse high-grade gliomas

**DOI:** 10.1007/s00280-026-04950-5

**Published:** 2026-09-17

**Authors:** Leire Balaguer-Lluna, Clàudia Resa-Parés, Lucas Brstilo, Alberto Gómez-Caballero, Mercè Baulenas-Farrés, Jacqueline Mohr, Yutian Hu, Rosario Aschero, Ana Rodríguez, Gaia Botteri, Eva Rodríguez, Lydia Li-Chen, Raúl Mañogil, Federica Marino, Nuria Martínez-Velasco, Alex Ruano, Ángela García-Pelayo, Auriane Giron, Jaume Mora, Cinzia Lavarino, Macarena Sánchez-Navarro, Meritxell Teixidó, Aida Ormazabal, Rafael Artuch, Nora Unceta, Paula Schaiquevich, Angel M. Carcaboso

**Affiliations:** 1 SJD Pediatric Cancer Center Barcelona, Hospital Sant Joan de Deu, Barcelona, 08950 Spain; 2 Institut de Recerca Sant Joan de Deu (IRSJD), Barcelona, 08950 Spain; 3 Unit of Innovative Treatments, Hospital de Pediatría JP Garrahan, Buenos Aires, 1245 Argentina; 4 National Scientific and Technical Research Council, CONICET, Buenos Aires, 1425 Argentina; 5 Department of Analytical Chemistry, Faculty of Pharmacy, University of the Basque Country (UPV/EHU), Vitoria-Gasteiz, 01006 Spain; 6 Hoffman-La Roche, Basel, 4058 Switzerland; 7 Pathology Department, Hospital Sant Joan de Deu, Barcelona, 08950 Spain; 8 Institute for Research in Biomedicine (IRB Barcelona), The Barcelona Institute of Science and Technology (BIST), Barcelona, 08028 Spain; 9 Clinical Biochemistry Department, Hospital Sant Joan de Deu, Barcelona, 08950 Spain; 10 Centre for Biomedical Research on Rare Diseases (CIBER-ER), Instituto de Salud Carlos III, Barcelona, 08950 Spain; 11 SJD Pediatric Cancer Center, Hospital Sant Joan de Deu, Santa Rosa 39-57, Esplugues de Llobregat, Barcelona, 08950 Spain

**Keywords:** Pediatric-type diffuse high-grade glioma (pHGG), Diffuse midline glioma (DMG), PI3K/AKT inhibitors, Inavolisib (GDC0077), CNS pharmacokinetics, *PIK3CA*-mutant xenografts

## Abstract

**Purpose:**

Pediatric-type diffuse high-grade gliomas (pHGG) are aggressive, largely incurable malignancies often characterized by PI3K/AKT pathway activation. This study investigated whether the PI3K inhibitor inavolisib and the AKT inhibitor ipatasertib could achieve therapeutic concentrations in the central nervous system (CNS) and demonstrate efficacy against *PIK3CA*-mutated pHGG.

**Methods:**

A panel of 12 pHGG cell lines with diverse PI3K/AKT genetic aberrations was screened for sensitivity to inavolisib and ipatasertib. Pharmacokinetic (PK) modeling was performed in mice to determine brain-to-plasma exposure ratios following oral administration (50 mg/kg inavolisib and 100 mg/kg ipatasertib). Finally, the in vivo efficacy of both inhibitors was evaluated using intracranial *PIK3CA*-mutated pHGG xenograft models, with survival and cerebrospinal fluid (CSF) circulating tumor DNA (ctDNA) levels as primary endpoints.

**Results:**

All 12 cell lines exhibited PI3K/AKT alterations. The *PIK3CA* H1047R-mutated line (HSJD-DIPG-007) was the most sensitive in vitro (IC_50_ = 0.016 µM for inavolisib; 0.52 µM for ipatasertib). PK modeling revealed geometric mean brain-to-plasma ratios of 0.07 for inavolisib and 0.20 for ipatasertib, with model-predicted maximum brain concentrations of 0.69 µM and 0.93 µM, respectively. In vivo, inavolisib significantly extended survival (*P* = 0.0070) and increased CSF ctDNA release (*P* = 0.0317). In contrast, ipatasertib did not demonstrate significant in vivo activity.

**Conclusion:**

These findings highlight the therapeutic potential of inavolisib for treating *PIK3CA*-mutated pHGG. Despite the blood-brain barrier, inavolisib achieved sufficient CNS exposure to prolong survival in preclinical models, whereas ipatasertib efficacy did not translate from in vitro to in vivo settings.

**Supplementary Information:**

The online version contains supplementary material available at https://doi.org/10.1007/s00280-026-04950-5.

## Introduction

Pediatric-type diffuse high-grade gliomas (pHGG) represent a group of highly aggressive malignancies of the central nervous system (CNS) [1]. Within the current WHO classification of CNS tumors, the diffuse midline glioma (DMG), H3 K27-altered, is the most prevalent molecular subclass, accounting for 80% of cases [2]. These tumors are primarily driven by somatic mutations in genes encoding histone H3.3 (H3F3A) or H3.1 (*HIST1H3B*, *HIST1H3C*), typically resulting in a lysine-to-methionine substitution at position 27 (K27M) [3, 4]. The most frequent clinical manifestation of DMG is diffuse intrinsic pontine glioma (DIPG), which originates in the brainstem. Most pHGG remain incurable and, for patients with DMG, surgical resection is clinically unfeasible due to the profuse infiltration of the tumor into vital neuroanatomical structures.

Since the discovery of oncogenic H3 K27 alterations in 2012, direct targeting of these mutations has remained clinically elusive, prompting research into alternative molecular vulnerabilities [5]. A primary candidate is the signaling axis involving receptor tyrosine kinases (RTK), phosphatidylinositol 3-kinase (PI3K), and protein kinase B (AKT). This pathway is constitutively activated by genomic alterations in 60% of pHGG [6]. For instance, approximately 10% of DMG cases harbor mutations in *PIK3CA*, the gene encoding the class I PI3K α isoform (p110α) [7]. While all three components of the RTK/PI3K/AKT pathway are targetable with small-molecule inhibitors developed over the last two decades [8–10], early clinical trials utilizing RTK inhibitors such as imatinib, vandetanib, and dasatinib failed to demonstrate significant activity in patients with pHGG. This lack of efficacy has been partially attributed to poor CNS drug penetration and distribution [11, 12].

The penetration of small molecules into the brain is commonly estimated using the brain-to-plasma area under the concentration-time curve (AUC) ratio. This assessment can be further refined by considering the protein-unbound fractions (representing the free molecules capable of traversing into brain tissue) and the ratio of unbound concentrations in the brain and plasma at steady state [13]. In most diffuse pediatric gliomas, the blood-brain barrier (BBB) remains largely intact [14]. This integrity contributes to the relatively low brain-to-plasma ratios observed in murine models for earlier inhibitors, such as 0.03 (i.e., 3%) for imatinib [15], 0.2 for vandetanib [16], and 0.1 for dasatinib [17].

More recently, the dual PI3K/mTOR inhibitor paxalisib demonstrated superior CNS penetration, with brain-to-plasma ratios exceeding 1 in rats and mice [18]. Paxalisib has since transitioned to clinical evaluation in patients with adult gliomas [19] and pHGG [20], where it has shown signs of activity in specific cohorts and achieved clinically relevant concentrations of approximately 1 µM in both brain and tumor tissue [19, 20]. This molecule continues in clinical evaluation in pHGG, while others, such as the AKT inhibitors, remain relatively unexplored.

In this study, we evaluated the pharmacology and CNS pharmacokinetics (PK) of two targeted inhibitors of the PI3K/AKT pathway. The first, inavolisib (GDC-0077), is a selective inhibitor of the PI3K p110α subunit and is an FDA-approved treatment for patients with metastatic *PIK3CA*-mutated breast cancer, following its landmark approval for use in combination therapies [21, 22]. Ipatasertib (GDC-0068), a selective ATP competitive pan-AKT inhibitor [23], underwent extensive evaluation in phase 3 clinical trials for metastatic prostate cancer [24]. However, its development was clinically discontinued after results fell below expected activity and safety thresholds. To date, neither inavolisib nor ipatasertib have been sufficiently tested in preclinical pHGG models and remain unassessed in clinical trials involving children with pediatric cancer. Crucially, their distribution within the CNS and diffuse high-grade gliomas is not entirely understood [25, 26]. The initial purpose of this work was to systematically assess the presence of RTK/PI3K/AKT alterations across a panel of 12 established pHGG cell lines. We then investigated the CNS disposition and anticancer efficacy of both inavolisib and ipatasertib in vivo, utilizing intracranial pHGG xenograft models with *PIK3CA* mutations, which we expected to be sensitive to inavolisib.

## Materials and methods

### Cell models

Primary cancer cell models, all pHGG (Supplementary Table S1), were established in our previous work at Hospital Sant Joan de Deu (HSJD, Barcelona, Spain) and cultured as tumorspheres in serum-free, B27-supplemented medium (pHGG medium) [27]. We obtained the human cerebral microvascular endothelial cells hCMEC/D3 from Sigma-Aldrich (Saint Louis, MO, USA) and cultured them in endothelial cell growth medium (Promocell, Heidelberg, Germany).

### Intracranial xenografts

We performed animal experimentation according to the Institutional and European guidelines (EU Directive 2010/63/EU) and with the approval of the local animal care and use committee from the University of Barcelona, Spain (protocol 135/11). We used 4-week-old athymic nude mice, all female (Inotiv, Barcelona, Spain). For xenografting, we inoculated 5 × 10^5^ HSJD-DIPG-007 (abbreviated DIPG-007) or HSJD-GBM-001 (GBM-001) cells [27], dispersed in 5 µL matrigel (BD Biosciences, San Diego, CA, USA), in the fourth ventricle of the brain. For this procedure, mice were anesthetized and immobilized in a stereotaxic apparatus (Stoelting, Wood Dale, IL), as previously described [27].

### Inhibitors

Ipatasertib (GDC-0068) and inavolisib (GDC-0077) were generously provided by Hoffmann-La Roche Ltd. (Basel, Switzerland). For in vitro assays, we prepared stock solutions in dimethyl sulfoxide (DMSO, Sigma-Aldrich) and stored them at -20 °C until use.

### Whole exome sequencing (WES)

Details for the extraction of DNA of cell lines, genomic DNA library and analysis are published [27].

### Immunofluorescence

For the detection of phosphorylated AKT (pAKT), we used 6 μm sections of formalin-fixed paraffin-embedded (FFPE) tumorspheres. We used pH 6 sodium citrate for antigen retrieval. We blocked with Novolink Polymer Protein Block (RE7200-CE, Leica Biosystems, Barcelona, Spain) for 45 min at room temperature (RT) in a humid chamber and incubated overnight at 4 °C with rabbit primary antibody for pAKT (#9271S, 1:50, Cell Signaling). The next morning, we washed the preparations with PBS and added the secondary antibody goat anti-rabbit conjugated with Alexa Fluor 488 (A11034, 1:1000, Thermo Fisher Scientific, Waltham, MA, USA) for 1 h at RT. We labeled cell membranes with wheat germ agglutinin (WGA, W11261, Thermo Fisher Scientific) and cell nuclei with 4′,6-diamidino-2-phenylindole (DAPI; 0.4 µg/ml; Merck, Darmstadt, Germany). We mounted the samples with Vectashield mounting medium (Vectorlabs, Newark, CA, USA) and obtained images with a fluorescence microscope (DM6B-Z, Leica Microsystems, Wetzlar, Germany).

### Antiproliferative activity of the inhibitors

For the quantification of metabolically active cells we used the compound [3-(4, 5-dimethylthiazol-2-yl)-5-(3-carboxymethoxyphenyl)-2-(4-sulfophenyl)-2 H-tetrazolium], MTS (Promega, Fitchburg, WI, USA). We plated 3000 cells per well on 96-well plates and incubated them at 37 °C and 5% CO_2_ for 24 h. Then, we added drugs at concentrations ranging 0-100 µM. The final concentration of DMSO in the 100 µM wells was maintained below 0.1% (v/v) to avoid vehicle-induced cytotoxicity. After 72 h, we detected the metabolization of MTS by measuring absorbance at 490 nm on a Multimode Plate Reader Infinite M nano (Tecan Group Ltd., Zurich, Switzerland). We calculated the half maximal inhibitory concentration, IC_50_, by building the concentration (log) *versus* response curves, with a variable slope (four-parameter) model, using Prism 10 software (GraphPad, La Jolla, CA, USA).

For the colony formation assay, we plated 5000 cells per well on 6-well plates coated with 2.5% matrigel and incubated overnight at 37 °C and 5% CO_2_. The next day, we treated the cells with the PI3K/AKT inhibitors at concentrations ranging 0.01-10 µM for 48 h. After treatment, we washed with fresh pHGG medium and incubated the cells for 10 days. We fixed the cell colonies with 4% paraformaldehyde (PFA, Sigma-Aldrich) and stained them with Coomassie (Bio-rad, Hercules, CA, USA). We quantified the colonies using iBright software (Thermo Fisher Scientific). These experiments were repeated three times.

### Endothelial cell tube formation assay

Pro-angiogenic signals secreted by DIPG-007 cells induce the formation of complex tubular structures in endothelial cells [28]. To address whether PI3K/AKT inhibitors abolished such effect, we coated 24-well cell culture plates with Geltrex (12760021, Thermo Fisher Scientific) and plated 10^5^ hCMEC/D3 cells in endothelial cell culture medium, pHGG medium, or DIPG cell culture supernatants (DIPG-007) in the presence or absence of 1 or 10 µM inavolisib or ipatasertib. After 18 h, we analyzed four fields from each condition with the Angiogenesis Analyzer plugin for ImageJ (National Institutes of Health, Bethesda, MD, USA). We measured the total length and counted the number of junctions of the new vascular network. Finally, we immunostained endothelial cells with primary antibody rabbit anti-human-CD31 (ab32457, 1:200, Abcam, Cambridge, UK) and secondary anti-rabbit-Alexa-594 (A21207, 1:1000, Invitrogen). We obtained the fluorescent images using the Leica THUNDER Imager Live cell and 3D Assay software (Leica Microsystems).

### Immunoblotting

We lysed samples using lysis buffer containing Tris-HCl pH 7.4 0.5 mM, NaCl 300 mM, 1% Triton 100X, EDTA 0.5 M, ortovanadate 100 mM, sodium fluoride 100 mM, sodium pyrophosphate 100 mM (all from Sigma-Aldrich), and EDTA-free protease inhibitor (11836170001, Sigma-Aldrich). We quantified proteins with the Bradford protein assay (Sigma-Aldrich) and loaded 40–70 ng of sample in 10–15% polyacrylamide gels. After electrophoresis, we transferred proteins to nitrocellulose membranes and blocked them with tris-buffered saline containing 5% bovine serum albumin (BSA, Sigma-Aldrich). Primary antibodies were PDGFRα (#3174), EGFR (#4267), IGF-1Rβ (#9750), AKT (#9272), phospho-AKT (Ser473) (pAKT, #4060), proline-rich AKT substrate of 40 kDa (PRAS40, #2691), phospho-PRAS40 (pPRAS40, Thr246, #2997), S6 ribosomal protein (S6, #2217), pS6 (Ser240/244; #5364), phospho-ERK (pERK, #4370), ERK (#4695), cleaved caspase-3 (#9664), cleaved caspase-7 (#8438), cleaved poly-ADP-ribose polymerase (cPARP, #5625), glyceraldehyde-3-phosphate dehydrogenase (GAPDH, #97166) (all from Cell Signaling), α-tubulin (T6199, Sigma-Aldrich), and lamin β1 (ab16048, Abcam). All primary antibodies were diluted 1:1000 and incubated overnight at 4 °C. Secondary antibodies were fluorescent-dye conjugated goat anti-rabbit and goat anti-mouse (926-32211 and 926-68070, LI-COR, Biosciences, Lincoln, NE, USA), diluted 1:5000 and incubated on membranes for 1 h at RT. Fluorescence signal was detected in the Odyssey CLx Imaging System (LI-COR). We quantified the obtained bands with ImageJ software.

### Detection of cPARP by flow cytometry

We treated one million DIPG-007 cells with inavolisib (0.01, 0.1, or 1 µM) or ipatasertib (0.1, 1, 5, or 10 µM), for 24 h, and fixed them with ethanol 70% overnight at -20 °C. Then, we washed with PBS and incubated for 30 min with PBS containing 1% inactivated fetal bovine serum (Thermo Fisher Scientific) and 1% BSA, on ice. Next, we incubated the cells with anti-cPARP (#5625, 1:400, Cell Signalling) for 20 min at 4 °C, and with secondary antibody anti-rabbit-Alexa 488 (ab150077, 1:500, Abcam) for 20 min at 4 °C in the dark. We used the NovoCyte flow cytometry system (ACEA Biosciences, San Diego, CA, US) for detection and analysed the percentage of apoptotic cells using the NovoExpress software (ACEA Biosciences).

### CNS drug distribution and PK modeling in mice

We suspended inavolisib powder in 0.5% (v/v) Tween 20 and 0.25% (w/v) carboxymethylcellulose (CMC) to a final concentration of 15 mg/mL. We administered 50 mg/kg inavolisib to 30 athymic nude mice, using an oral gavage. Mice were randomized into independent time-point cohorts and dosed using a staggered schedule (up to 10-min intervals for early time points) to ensure precise post-dose sampling windows. Body weight stratification was unnecessary due to the high homogeneity (identical age, sex, and vendor batch) and narrow weight range of the animals. At 0.25, 0.5, 1.0, 2.0, 6.0, 12.0, 18.0, and 24.0 h after drug administration we selected three mice randomly for sample collection. Under ketamine-xylazine anesthesia we obtained one blood sample from the retro-orbital plexus and isolated the plasma by centrifugation. Then, we immobilized the animals in a stereotaxic instrument and collected the CSF from the cisterna magna, as reported [27]. Finally, we euthanized the animals by decapitation and obtained brain samples.

For ipatasertib, we performed two PK experiments. In the first, 10 mice received 10 mg/kg of the drug, dissolved in saline solution, through a bolus injection in the tail vein. We obtained a maximum of two blood samples from each mouse, the first from the retro-orbital plexus under isoflurane anesthesia, and the second from the heart under ketamine-xylazine, followed by euthanasia. Sampling times were 0.5, 1.0, 2.0, 6.0, and 18.0 h after administration. For the second experiment, we administered an oral dose of 100 mg/kg ipatasertib (suspended in 0.25 CMC and 0.5% Tween 20) to 45 mice, 20 of which had intracerebral DIPG-007 xenografts (day 52 post-inoculation; time in which the infiltrative tumor is established, but still far away from terminal stage) [27]. We obtained a maximum of two blood samples from each mouse, as described above. After euthanasia, we collected brain samples at time points 0.25, 0.5, 1.0, 2.0, 6.0, 12.0, 18.0, and 24.0 h. We stored all samples at -80 °C until drug analysis.

We quantified drugs by liquid chromatography-tandem mass spectrometry (LC-MS/MS) (Supplementary Method S1).

For PK modeling, we designed a population-based study in which we included all the samples obtained per animal. We analyzed data by means of population PK modeling with MONOLIX Suite 2024R1 (Lixoft SAS, a Simulations Plus company) using the Stochastic Approximation Expectation Maximization (SAEM) algorithm combined with a Markov Chain Monte Carlo (MCMC) approximation. We assumed inter-individual and residual variability were log-normally and normally distributed, respectively, and tested different residual models. Throughout the modeling process, we tested model selection based on the change in the objective function value (OFV), the Akaike information criteria (AIC), the Bayesian information criteria (BIC), the precision of parameter estimates, and goodness-of-fit plots. Data below the limit of quantification were left-censored and analyzed following the M4 method described by Beal [29].

We used empirical Bayesian estimations to calculate the individual model parameters for each animal, which were subsequently processed in Simulx^®^ (Lixoft, Simulations Plus, Antony, France) to simulate complete plasma, brain, and CSF concentration-time profiles. Exposure in each compartment (AUC_plasma_, AUC_brain_, and AUC_CSF_), as well as the maximum concentration (C_max_) and the corresponding time at which C_max_ occurred (T_max_), were derived from the simulated individual concentration-time profiles using Simulx.

### Anticancer activity in xenografts

For the inavolisib study, we assigned 21 mice bearing intracranial DIPG-007 xenografts to groups receiving either vehicle (*n* = 11) or 50 mg/kg inavolisib (*n* = 10) by oral gavage, once daily at post-inoculation days 32–36, 39–43, 53–57 and 60–64. For ipatasertib, we assigned 39 mice bearing DIPG-007 to three groups receiving vehicle (*n* = 14) or ipatasertib at dosages of 25 mg/kg (*n* = 15) or 100 mg/kg (*n* = 10). Doses were given orally once a day, at days 32–36, 39–43, 46–50, 53–57 and 60–64 after tumor inoculation. For ipatasertib, we evaluated efficacy in a second intracranial xenograft, GBM-001, with doses (25 mg/kg or vehicle) administered orally to groups of 10 mice at days 10–14, 17–21 and 24–28.

After finishing treatments, all animals were observed daily for tumor progression. We calculated the median survival of each group once all the animals achieved endpoint, which was defined as the day on which animals lost 20% of their body weight due to disease (using the maximum individual weight as a reference).

To evaluate markers of anticancer activity, such as the presence of apoptotic cPARP^+^ cells at the tumor site, we euthanized mice during treatments. For inavolisib, we performed an independent assay with mice bearing DIPG-007 xenografts receiving vehicle or drug (*n* = 5 for both). We euthanized all mice of each group, 2 h after the 14th dose of treatment (day 36, in this experiment), and collected CSF and brains (FFPE) for analysis. For ipatasertib, we obtained brain samples from the vehicle and 25 mg/kg groups (*n* = 5, each) of the above-mentioned survival assay, 2 h after the 20th dose (day 57).

### Quantification of the tumor burden in the mouse brain

To estimate the amount of human tumor in FFPE sections, we performed immunostaining of human cells (primary antibody anti-human nuclear antigen MAB4383, 1:200, Sigma) and droplet digital PCR (ddPCR; to count human *H3F3A* DNA copies), as we described elsewhere [27].

### Quantification of circulating tumor DNA (ctDNA)

First, we evaluated in vitro whether PI3K/AKT inhibitors induced the release of ctDNA to the culture medium. We plated 200,000 DIPG-007 cells per well, in six-well plates, and cultured them overnight. The next day, we treated half of the wells with 1 µM inavolisib, and added fresh pHGG medium to the other half. Four and nine days later, we counted the cells and collected the supernatants for ctDNA analysis of human *H3F3A* copies in the processed samples, as reported [27]. We applied the same method to quantify *H3F3A* copies in the mouse CSF.

### Statistics

Grouped data are presented as mean ± standard deviation (SD), or as geometric mean (GM) accompanied by the geometric SD or geometric coefficient of variation (geoCV%) for log-normally distributed PK parameters. We carried out statistical analyses using Graphpad Prism 10. For comparisons between two groups, we applied the Student’s t test or the Mann Whitney test (for non-normally distributed data). To compare more than two groups, we used the one-way ANOVA or the Kruskal-Wallis test with Dunn’s multiple comparison test correction. For comparisons of two variables in multiple groups, we applied the two-way ANOVA with Šidák’s multiple comparisons test. For all tests, *P* < 0.05 was considered significant.

## Results

### Alterations of the PI3K/AKT pathway in pHGG cells

**Fig. 1:**
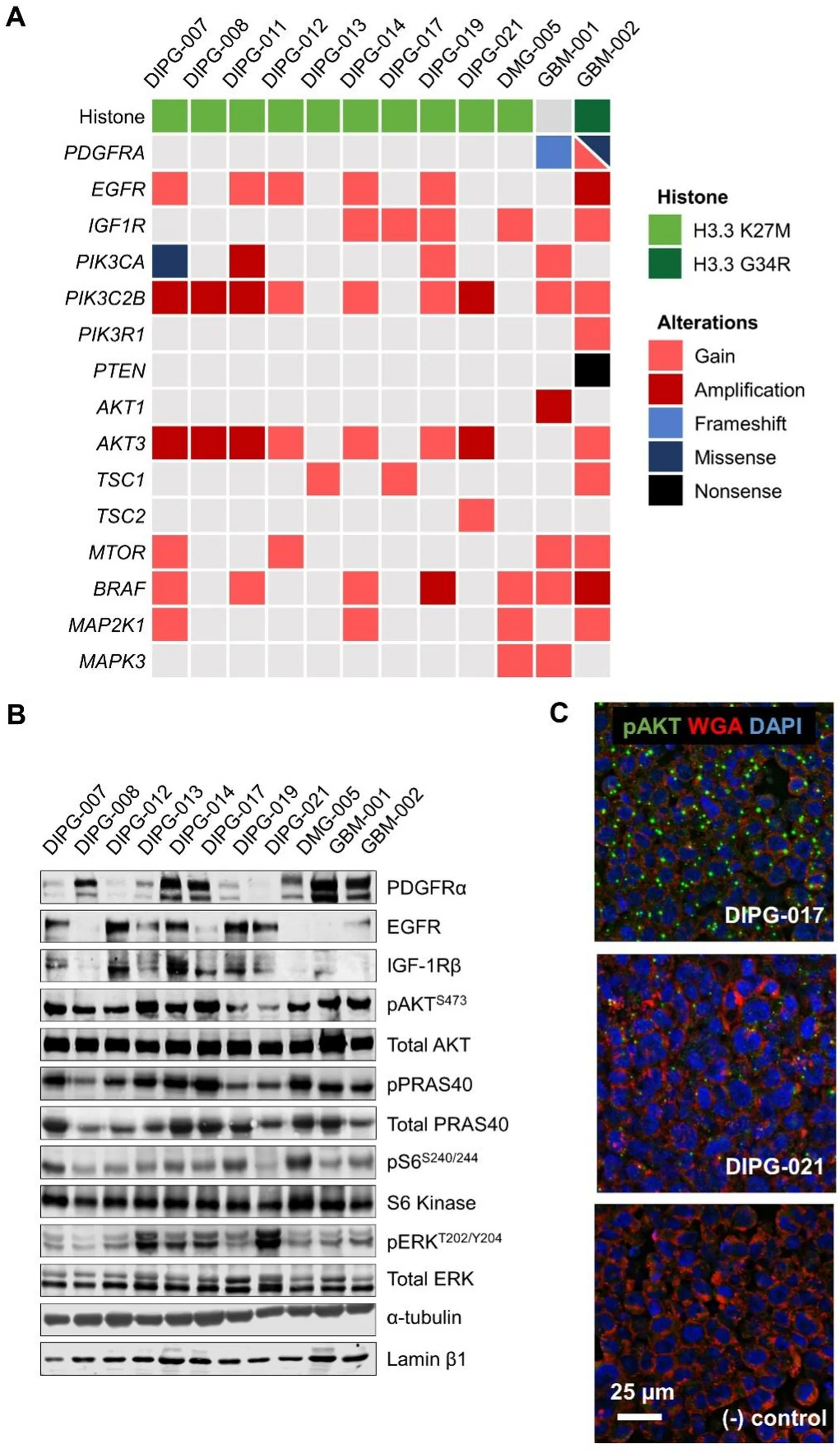
Analysis of the RTK-PI3K-MAPK axis in pHGG lines. (**A**) Main alterations found by WES analysis in genes related to the RTK/PI3K/AKT and MAPK/ERK pathways, with annotations of histone mutations, DNA copy-number alterations (gain, amplification and deletion) and single-nucleotide variants (frameshift, missense and nonsense mutations). (**B**) Expression of proteins involved in the RTK, PI3K/AKT and MEK/ERK nodes in pHGG models in culture. α-tubulin and lamin β1 were used as loading controls, for proteins ranging 30–175 kDa or above 175 kDa, respectively. (**C**) Immunofluorescence of pAKT (green) in DIPG models with the highest (DIPG-017) and lowest (DIPG-021) pAKT expression by immunoblotting. The cytoplasmic membrane was stained with WGA (red) and the nucleus with DAPI (blue). The negative (-) control was the DIPG-017 sample without adding primary antibody. For clarity purposes, we omitted the institutional preffix HSJD in the ID of the primary cultures

All the sequenced lines showed alterations in the RTK-PI3K-AKT axis (Fig. 1A). The complete set of non-synonymous variants and variant allele frequencies of the WES analysis are published elsewhere [27]. Among the genes encoding for RTK receptors we identified amplification of epidermal growth factor receptor (*EGFR*) in 50% of the samples, insulin-like growth factor 1 receptor (*IGF1R*) in 42%, and platelet-derived growth factor receptor alpha (*PDGFRA*) in 8% (one case). *PDGFRA* was mutated in the two lines derived from hemispheric tumors.

The p110β catalytic subunit (*PIK3C2B*) of PI3K was amplified or gained in 75% (nine) of the pHGG models (Fig. 1A). Four of them showed concomitant alterations in the p110α catalytic subunit PI3K (*PIK3CA* gene), such as the missense mutation *PIK3CA* H1047R found in DIPG-007 cells. Another line with *PIK3C2B* gain, HSJD-GBM-002 (GBM-002), carried a gain of the regulatory subunit gene (*PIK3R1*) and the nonsense mutation *PTEN* Q298* (Fig. 1A).

We included in the analysis alterations of the mitogen-activated protein kinase (MAPK) cascades, because the extracellular signal-regulated kinase (ERK) and the MAPK/ERK kinase (MEK) are frequently co-activated as a resistance mechanism when the PI3K pathway is inhibited with medicines [30, 31]. In our panel, we found the gene *BRAF* amplified in 17% samples and gained in 42%, *MAP2K1* (which encodes MEK) gained in 25%, and *MAPK3* (encoding ERK) gained in 12%, with one line, HSJD-DMG-005 (DMG-005), having alterations in the three mentioned genes (Fig. 1A).

Subsequently, we examined the activation of the RTK/PI3K/AKT signalling pathway using immunoblotting (Fig. 1B). The expression of RTK proteins PDGFRα and EGFR was mutually exclusive. Cells such as HSJD-DIPG-008, DMG-005, GBM-001 and GBM-002 showed high expression of PDGFRα and low or undetectable expression of EGFR or IGF-1Rβ. On the contrary, cells such as DIPG-007, HSJD-DIPG-012, HSJD-DIPG-013, HSJD-DIPG-019 and HSJD-DIPG-021 (DIPG-021) showed low PDGFRα and high EGFR and IGF-1Rβ expression. Phosphorylation of AKT at Ser473 was present in all the cultures (Fig. 1B). The protein levels of pAKT detected by immunoblotting coincided with the immunofluorescence signal in the cultured tumorspheres (Fig. 1C). PRAS40 protein, a direct effector of AKT, showed higher expression in those cells with high levels of activated pAKT. Similarly, pS6 was expressed by all the cultures (Fig. 1B). With regard to the MAPK pathway, ERK1/2 was substantially phosphorylated in the line DIPG-021, which may indicate a strong dependency on the MAPK pathway signalling (Fig. 1B).

### Inhibition of cancer cell proliferation, clonogenicity and angiogenesis

**Fig. 2:**
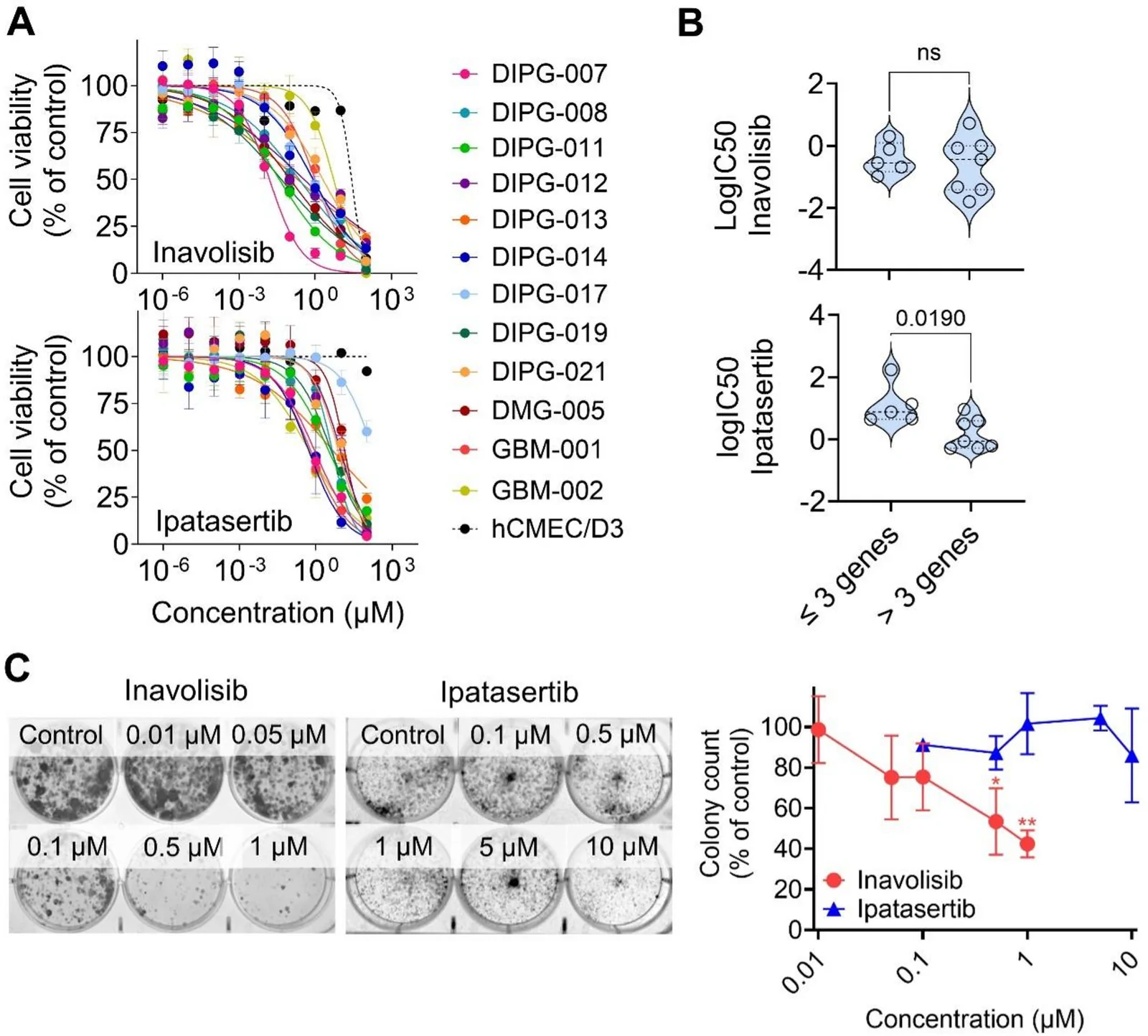
Inhibition of cancer cell proliferation and clonogenicity by inavolisib and ipatasertib. (**A**) Antiproliferative activity in pHGG models and human cerebral microvascular endothelial cells (hCMEC/D3). Dots and error bars are the mean ± SD of 3–6 replicates, and curves are the best fit using Graphpad. (**B**) LogIC50 values (violin plots and individual data) obtained for the pHGG models, grouped by the number of alterations in PI3K/AKT genes (≤ 3 genes or > 3 genes). (**C**) Representative images of a colony formation assay, with graphics representing the percentage of colonies with reference to the control condition (mean and SD of three independent experiments). *P = 0.0252 and **P = 0.0064, compared to untreated controls (one-way ANOVA with Dunnet’s multiple comparison test)

Ipatasertib and inavolisib inhibited the proliferation of most pHGG models included in the activity panel, with IC_50_ values in the range of 0.016–5.33 µM for inavolisib (median 0.32 µM) and 0.52–175 µM for ipatasertib (median 4.19 µM) (Fig. 2A, Supplementary Table S2). The viability of the brain endothelial cell line hCMEC/D3 was affected only by inavolisib, at concentrations above 10 µM (Fig. 2A). We found that pHGG cells bearing more than three alterations in PI3K/AKT genes were significantly more sensitive to ipatasertib, while this property did not affect the sensitivity to inavolisib, for which the *PIK3CA* H1047R-mutant model, DIPG-007, was the most sensitive line (Fig. 2B, Supplementary Table S2).

Inavolisib inhibited significantly the clonogenic capacity of DIPG-007 cells at concentrations above 0.5 μm, while ipatasertib did not induce such effect, even at 10 µM (Fig. 2C).

As we observed in previous work [28], pHGG cell culture supernatants favored the formation of complex vascular structures by brain microvascular endothelial hCMEC/D3 cells (Supplementary Fig. S1). The addition of inavolisib (1 and 10 µM) to the medium inhibited significantly the formation of such complex structures, while ipatasertib induced similar effects only at 10 µM (Supplementary Fig. S1).

### Pharmacodynamics of inavolisib and ipatasertib in pHGG cells

**Fig. 3:**
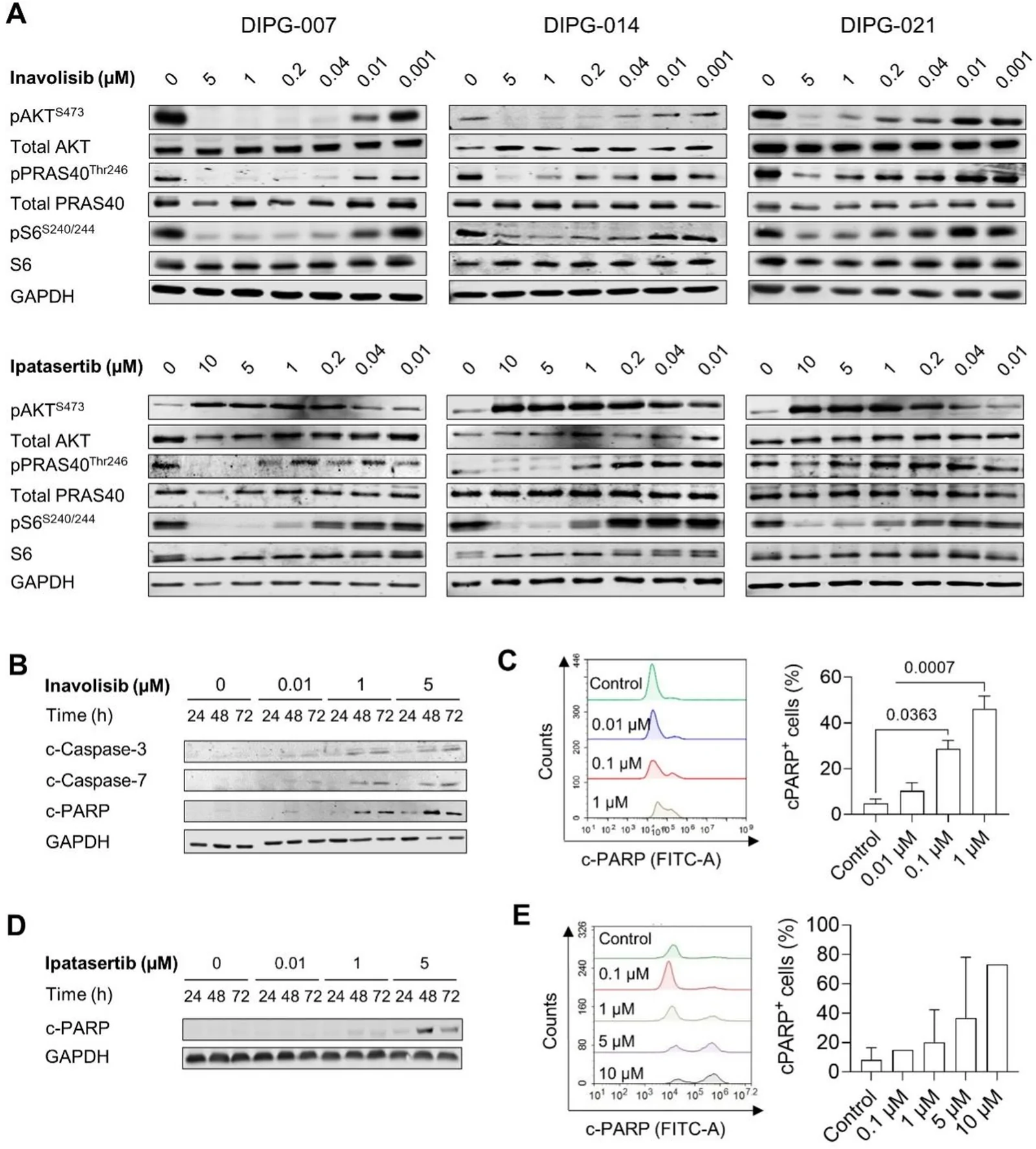
PI3K/AKT pathway inhibition and apoptotic response to inavolisib and ipatasertib in vitro. (**A**) Immunoblotting of downstream signalling proteins in response to inavolisib and ipatasertib treatment of DIPG-007, DIPG-014 and DIPG-021 cells. GAPDH was the loading control. (**B**) Immunoblotting of apoptotic-related proteins in DIPG-007 cells treated with inavolisib. GAPDH was the loading control. (**C**) Representative flow cytometry graphics and quantification of cPARP^+^ cells (mean ± SD of four experiments) in DIPG-007 cells treated with inavolisib. (**D**) Immunoblotting of cPARP in DIPG-007 cells treated with ipatasertib. GAPDH was the loading control. (**E**) Representative flow cytometry graphics and quantification of cPARP^+^ cells (mean ± SD of two experiments) in DIPG-007 cells treated with ipatasertib

We addressed the inhibition of the downstream PI3K/AKT cascade in pHGG. Phosphorylation of AKT (pAKT) at S473 was used as the readout for AKT activity, and pPRAS40 and pS6 were used as surrogate readouts for mTOR activity. Two of the cell lines included in the assay, DIPG-007 and HSJD-DIPG-014 (DIPG-014), were selected for having more than three alterations in RTK/PI3K/AKT genes, while the third line, DIPG-021, only had two. In the immunoblotting assay all the lines showed constitutive phosphorylation of AKT, PRAS40 and S6 in basal condition (Fig. 3A). Inavolisib potently inhibited pAKT, pPRAS40 and pS6 levels in a dose-dependent manner, with DIPG-007 being particularly sensitive to the drug (high inhibition of the pathway at 0.04 µM) and DIPG-021 being resistant at concentrations below 1 µM (Fig. 3A). For ipatasertib, we observed the expected accumulation of pAKT following treatment, explained by the ATP-competitive (active-site–directed) action of the drug, targeting the phosphorylated conformation of the protein AKT [23]. We also found downstream suppression of pPRAS40 and pS6 at concentrations above 1 µM. The inhibition of the pathway with ipatasertib was also more effective in DIPG-007 cells than in the other two lines (Fig. 3A).

Regarding apoptosis, the protein levels of cleaved caspases and cPARP and the number of cPARP^+^ cells increased after inavolisib treatment of DIPG-007 cells, particularly after 48 h, and at concentrations above 10 nM (Fig. 3B, C). We observed that the apoptotic effects of ipatasertib were restricted to concentrations above 1 µM (Fig. 3D, E).

### CNS distribution of inavolisib

**Fig. 4:**
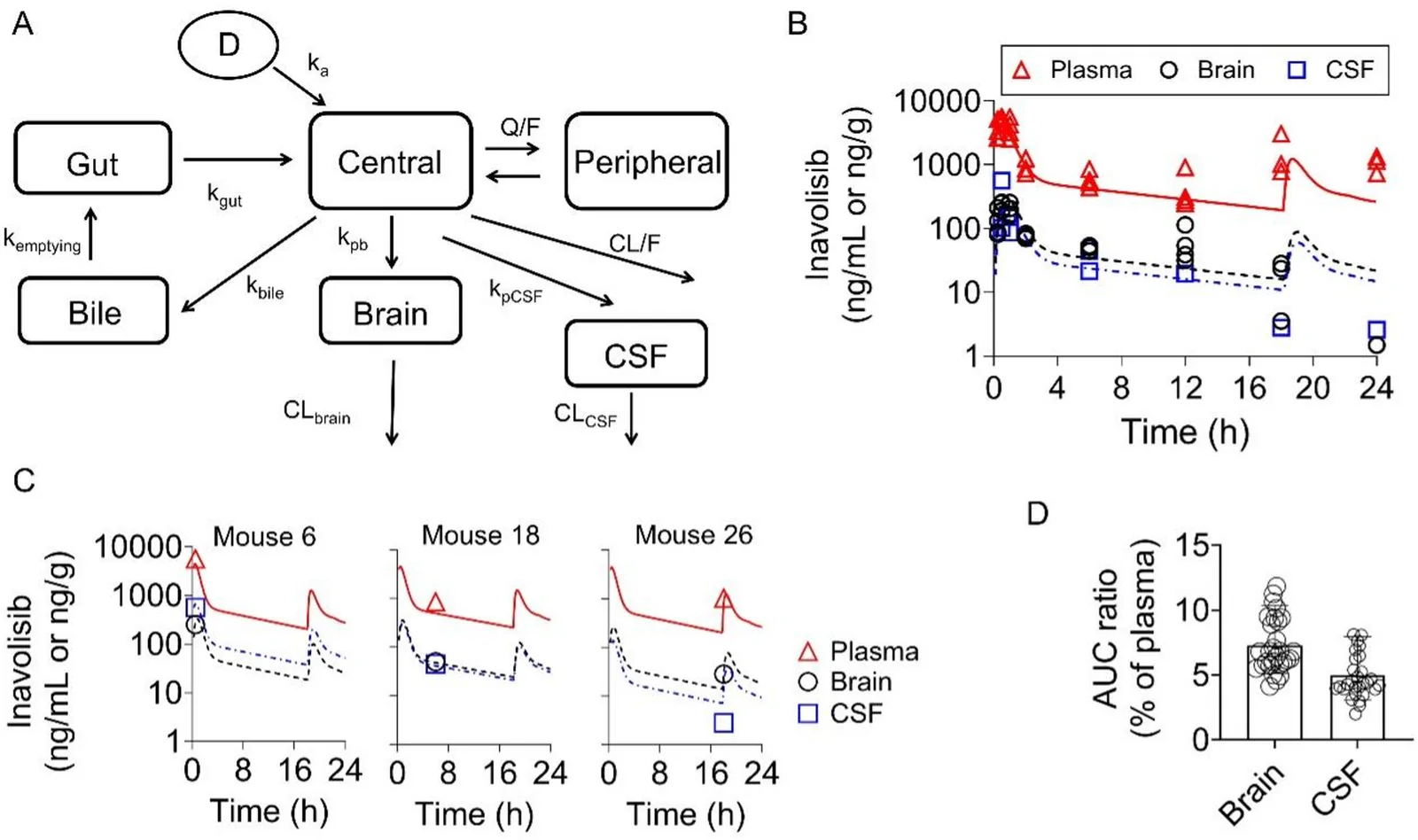
CNS distribution of inavolisib in athymic nude mice receiving one 50 mg/kg oral dose. **A** Schematic description of the compartmental model. The parameters CL, CL_brain_, CL_CSF_, k_a_, k_bile_, k_empt_, k_gut_, k_pb_, k_pCSF_ and Q, are defined in the main text. D: dose; F: bioavailability. **B** Inavolisib concentrations in plasma, brain and CSF. Lines are the best-predicted concentrations for a representative animal of the population. Individual symbols are the observed concentrations in plasma, brain and CSF. **C** Representative concentration-time profiles (experimental data and model-fitted curves) of inavolisib in plasma, brain and CSF of three individual mice. **D** Brain-to-plasma and CSF-to-plasma AUC ratios. Dots represent data from individual mice and columns are the geometric means with geometric SD

We implemented a sequential three-step analysis to build the model that best described the PK of inavolisib (Fig. 4A**)**. For this, we used plasma, brain, and CSF concentration *versus* time data from 30 mice receiving 50 mg/kg oral inavolisib. In the absence of the bioavailability (F) value for inavolisib in athymic nude mice, we estimated the PK parameters for absorption (Ka; constant rate of the absorption from the gut to the central compartment), distribution (Q/F; intercompartmental clearance) and elimination (CL/F; clearance) of inavolisib in plasma (step 1). We explored an alternative structural one-compartment model during model development and compared both models using diagnostic plots and information criteria (OFV, AIC, BIC), and precision of parameter estimates (Supplementary Table S3**)**. We observed that (i) the drug rapidly reached peak plasma concentrations with a calculated GM C_max_ of 4441 ng/mL (geoCV% = 2%) attained at 0.5 h (range: 0.4–0.6 h) after drug administration, and (ii) the concentration *versus* time profile in plasma showed the appearance of a secondary peak after approximately 18 h of drug dosing (Fig. 4B, parameter T_first_). Therefore, we included the process of enterohepatic reabsorption [32] in the population PK model to describe this phenomenon. The model included the parameters k_bile_, k_emptying_, and k_gut_ to describe the transfer rates between the central compartment and the bile, between the bile and the gut, and between the gut and the central compartment, respectively (Fig. 4A). K_emptying_ is a time-dependent rate constant that controls the emptying process, and we assumed k_gut_ = k_a_. Then, for the second and third steps of model building, we fixed the estimated parameters in step 1 and modeled inavolisib concentrations in brain and CSF, estimating the rate constants governing plasma-to-brain penetration (k_pb_), plasma-to-CSF penetration (k_pCSF_), drug elimination from brain (CL_brain_), and elimination from CSF (CL_CSF_) after inavolisib administration. All these processes were considered to follow first-order kinetics. We fixed the CSF volume of distribution (V_CSF_) to 0.036 mL, according to reference values in mice and considering a density of 1 g/mL for the brain tissue [33]. The equations for the final model of inavolisib are provided in Supplementary Material S1. The calculated population estimates are in Supplementary Table S4, corresponding to the mean values of the PK parameters obtained from all animals included in the studies. Model performance was supported by goodness-of-fit plots analyses, which demonstrated good agreement between observed and predicted concentrations (Supplementary Fig. S2A-L). The model adequately described the experimental concentration-time profiles in all sampled tissues, as depicted for three individual animals in Fig. 4C. The GM brain-to-plasma AUC ratio was 0.07 (geoCV% = 36%) and the GM CSF-to-plasma AUC ratio was 0.05 (geoCV% = 47%) (Fig. 4D).

We observed a rapid transfer of inavolisib from plasma to the brain, as the estimated GM C_max, brain_ of 280 ng/g (i.e., 0.69 µM; geoCV% = 29%) was attained at 0.7 h (range: 0.6–1.1 h). In line with these findings, inavosilib C_max_ in the CSF was also attained at 0.7 h (range: 0.6–1.1 h). Inavolisib C_max_ in the CSF was 191 ng/mL (i.e., 0.47 µM; geoCV% = 43%). Drug exposure in the CSF was lower, compared to that attained in the brain of the animals (GM AUC_CSF_/AUC_brain_ = 0.66; geoCV% = 24%).

### CNS distribution of ipatasertib

**Fig. 5:**
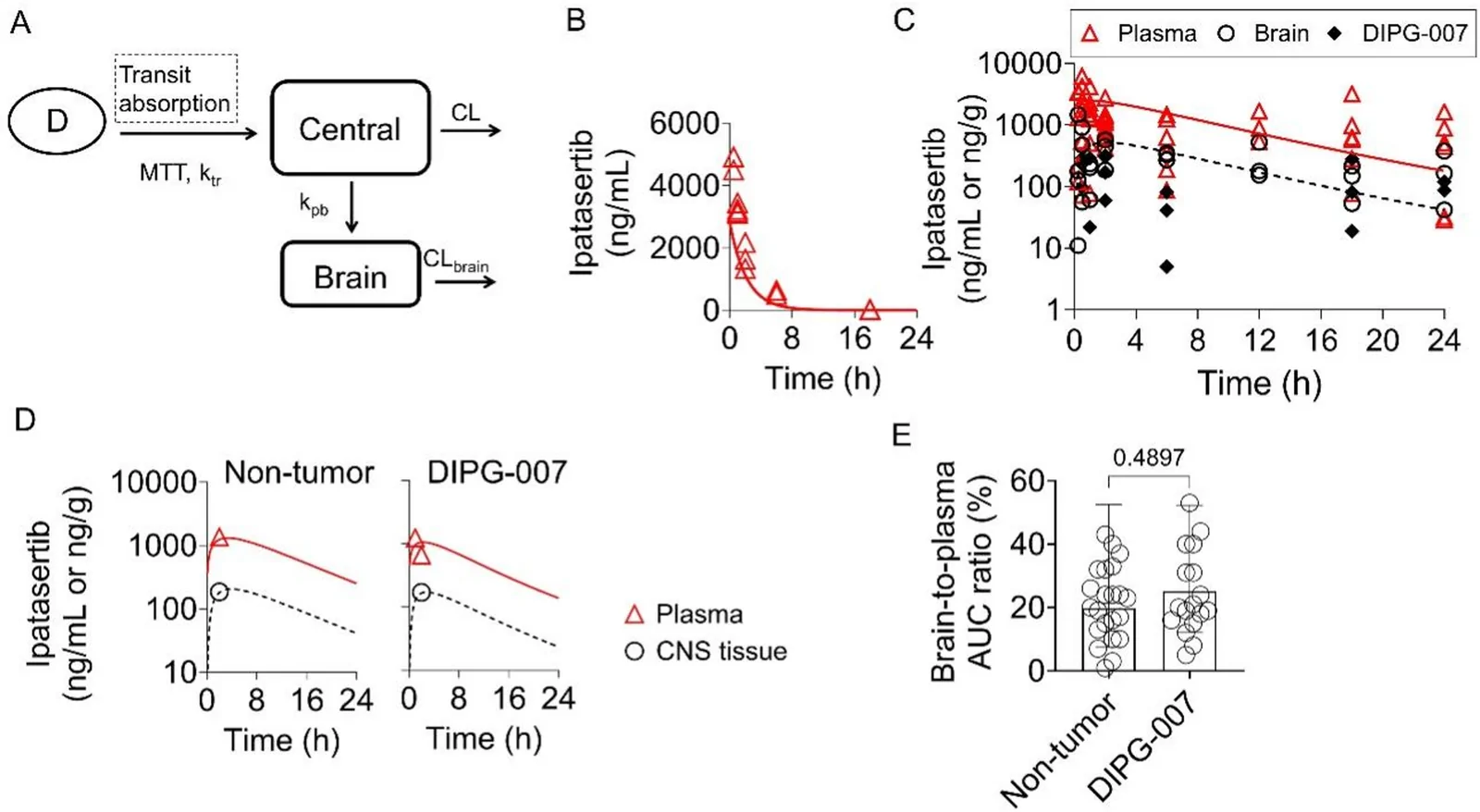
CNS distribution of ipatasertib in mice receiving one 100 mg/kg oral dose. **A** Schematic description of the compartmental model. The parameters CL, CL_brain_, k_a_, and k_pb_ are defined in the main text. D: dose; K_tr_: transit rate constant; MTT, mean transit time. **B** Ipatasertib concentrations in plasma of mice receiving 10 mg/kg of the drug through a bolus injection in the tail vein. The line is the best-predicted concentration for a representative animal of the population. Individual symbols are the observed concentrations. **C** Ipatasertib concentrations in plasma and CNS in mice without tumors (brain – no tumor) and with tumors (brain – DIPG-007). Lines are the best-predicted concentrations for a representative animal of the population. Individual symbols are the observed concentrations in the analyzed samples. **D** Representative concentration-time graphs (experimental data and model-fitted curves) of ipatasertib concentration in plasma and CNS tissue of individual mice without tumors (non-tumor) or with intracerebral DIPG-007. **E** CNS-to-plasma AUC ratios of mice without tumors (non-tumor) or with intracerebral DIPG-007. Dots are data from individual mice and columns are the geometric means with geometric SD

Our PK analysis approach led to the model shown in Fig. 5A. First, we pooled intravenous and oral PK data, and estimated absorption, distribution, elimination and bioavailability parameters simultaneously. The transit-compartment absorption model performed better than the first-order absorption model (Supplementary Table S5). The second part of the PK analysis (step 2) was similar to the one described for inavolisib. The final equations are in Supplementary Material S2 and the model successfully fitted ipatasertib concentration *versus* time data in the plasma and the CNS of mice without tumors and also of mice bearing intracranial DIPG-007 xenografts (Fig. 5B, C). A one-compartment model with linear elimination and transit-compartment absorption best described the plasma concentration-time data following intravenous and oral administration, with the early distribution phase and C_max_ being model-predicted due to the absence of samples prior to the initial 0.5 h time point. The mean population parameters and goodness-of-fit plots for the final model of ipatasertib are detailed in Supplementary Table S6 and Supplementary Figs. S3A-H and S4A-D. The estimated oral bioavailability of ipatasertib was 23% (relative standard error: 19.2%), with moderate interindividual variability (40.5%). We found a relatively slow absorption process into the central compartment, with GM plasma C_max_ of 1975 ng/mL (geoCV% = 73%), attained after 1.9 h (range: 0.5–6.5 h) of oral drug administration (Fig. 5C). The drug was rapidly distributed from the central compartment (plasma) to the brain, as reflected by the similar time to achieve the C_max_ (median T_max_ = 2.7 h; range: 0.9–6.8 h) in this tissue. However, the fraction of ipatasertib that penetrated into the brain from plasma was low and highly variable, with GM C_max, brain_ = 427 ng/g (geoCV% = 73%), equivalent to 0.93 µM, and a GM brain-to-plasma AUC ratio of 22% (geoCV% = 73%). The GM brain-to-plasma AUC ratios were similar in mice without tumors (20%; geoCV% = 126%) and in mice with DIPG-007 (25%; geoCV% = 83.8%), indicating comparable brain penetration between groups. Despite similar brain penetration, substantial between-animal variability was observed in both groups (Fig. 5D, E).

### Activity in DIPG xenografts

**Fig. 6:**
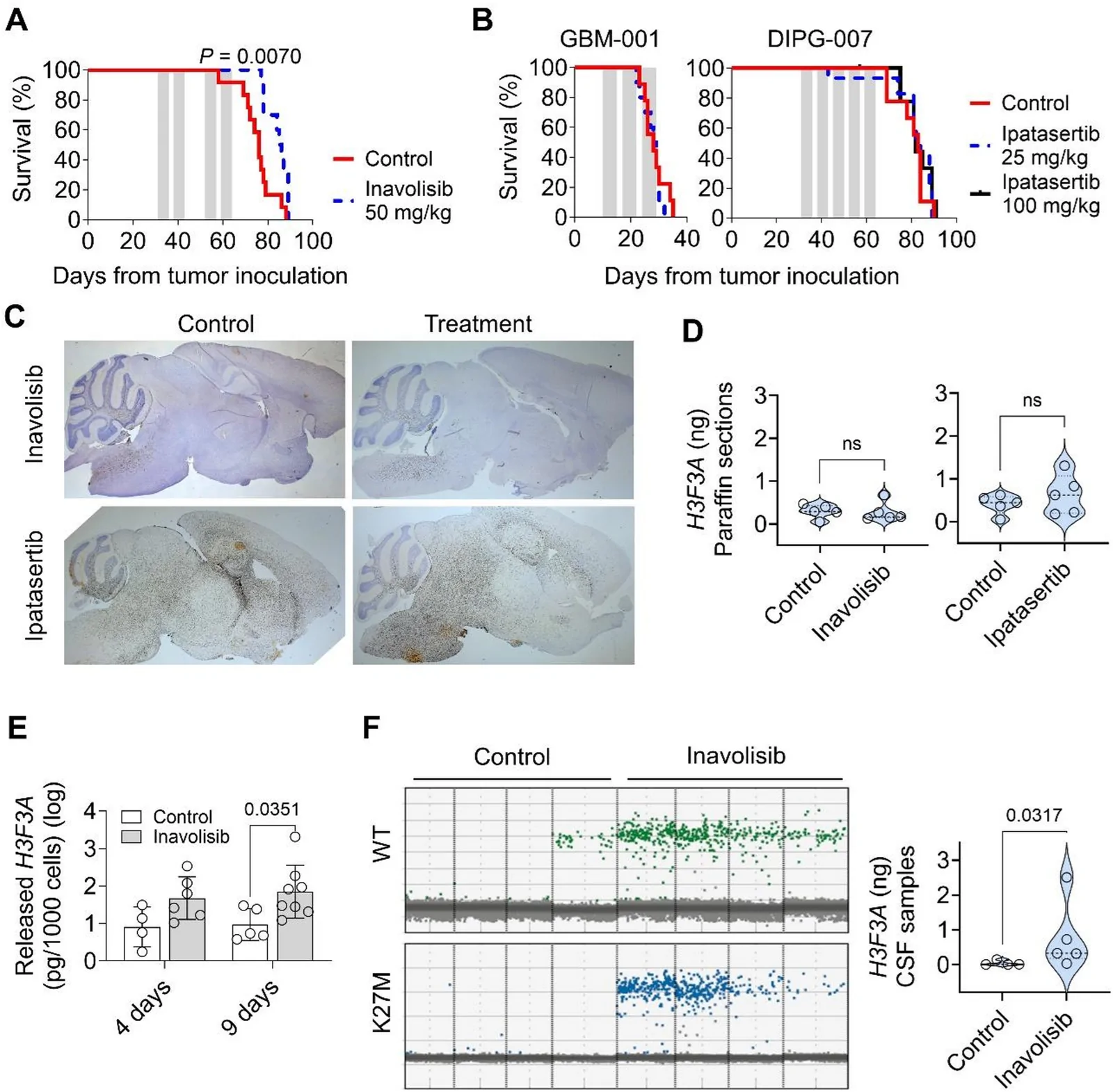
In vivo activity of inavolisib and ipatasertib in mice bearing intracranial pHGG xenografts. (**A**) Kaplan Meier curves of the survival experiment in DIPG-007-bearing mice treated with 50 mg/kg inavolisib, compared to vehicle-treated controls. (**B**) Kaplan Meier curves of GBM-001 and DIPG-007-bearing mice treated with 25 or 100 mg/kg ipatasertib. (**C**) Representative panoramic photographs of mouse brains bearing DIPG-007 xenografts obtained at the end of treatment. Human cells are those whose nuclei are stained in brown. Tumor load was higher for the ipatasertib study due to sampling times (36 and 57 days after tumor inoculation for inavolisib and ipatasertib, respectively). (**D**) Quantification of human *H3F3* gDNA (ng) in mouse brain FFPE slides obtained from DIPG-007-bearing mice treated with inavolisib and ipatasertib, or from untreated mice. Samples were analyzed after the 14th dose of treatment of inavolisib and after the 20th of ipatasertib. (**E**) Release of human *H3F3A* from DIPG-007 cells following treatment with 1 µM inavolisib. Values are expressed as the *H3F3A* release (pg) per 1000 alive cells in culture. Dots are individual replicates and columns are mean and SD. For statistical analysis we used the two way ANOVA with Šídák’s multiple comparisons test. (**F**) Quantification of human *H3F3A* in the CSF of DIPG-007 xenografts after the 14th dose of inavolisib treatment. Graphics show the droplets for the alleles *H3F3A* K27M and *H3F3A* WT from four representative samples of each group, treatment and control. The violin plot graphic is the comparison (t test) of the mean values of *H3F3A* (ng) obtained for each group, being the dots data from individual mice

The median survival of the intracranial DIPG-007 mouse xenografts treated with inavolisib was 86 days, significantly longer compared to the control mice, which survived 76 days (*P* = 0.0070) (Fig. 6A). Ipatasertib treatment did not achieve therapeutic benefit in DIPG-007 and GBM-001 xenografts, regardless of the dose (Fig. 6B). Neither of the treatments induced a significant decrease in the infiltrating tumor load during treatment, as measured by immunohistochemistry (Fig. 6C and Supplementary Fig. S5) or ddPCR in paraffin sections (Fig. 6D). We did not detect differences in the count of apoptotic (cPARP^+^) cells in the brain xenograft in inavolisib-treated mice and controls (Supplementary Fig. S6A, B). Inavolisib treatment of DIPG-007 cells in culture increased ctDNA in the supernatants (Fig. 6E). We also observed a significant increase of ctDNA in the CSF obtained from mice treated with inavolisib (Fig. 6F).

## Discussion

Aberrations in the RTK/PI3K/AKT pathway and high levels of phosphorylated AKT are common molecular events in pHGG and other pediatric cancers, contributing to increased malignancy [34]. In this study, we demonstrate that the PI3K inhibitor inavolisib exhibits in vivo therapeutic activity against *PIK3CA*-mutated diffuse pediatric glioma. This activity is supported by modest but sufficient CNS penetration, maintaining brain concentrations above 50 ng/g (0.12 µM) for at least 2 h. Conversely, we found that insufficient CNS distribution precludes the AKT inhibitor ipatasertib from being a viable candidate for pHGG treatment.

Our analysis identified several genomic alterations leading to RTK/PI3K/AKT signaling activation in pHGG lines that are highly representative of the clinical disease. These findings align with those reported by the research group led by Matthew Dun, who utilized CRISPR/Cas9 loss-of-function screening to identify a significant genetic dependency on *PIK3CA* and mTOR for pHGG cell proliferation in vitro [35]. Their work highlighted the therapeutic potential of the PI3K/mTOR inhibitor paxalisib, with a one-month course of oral paxalisib monotherapy extending the median survival of aggressive DIPG xenografts (including our challenging *H3F3A*/*ACVR1*/*PPM1D*/*PIK3CA*-mutant HSJD-DIPG-007 model) by 10–14 days [35]. In the present study, we selected inavolisib over paxalisib due to its more targeted mechanism of action against *PIK3CA*-mutated cancers [36]. However, whether this specificity results in superior disease control or a more favorable safety profile compared to paxalisib remains to be determined. Notably, clinical trials have shown that PI3K/AKT inhibitors, including both paxalisib and inavolisib, induce hyperglycemia in approximately 10% of patients [19, 22].

The pronounced in vitro activity of inavolisib and ipatasertib in our DIPG-007 model may be attributed to the H1047R *PIK3CA* hotspot mutation. This mutation enhances the lipid kinase activity of p110α, inducing a gain of function in PI3K and subsequently increasing pAKT levels [37]. For instance, inavolisib has been shown to inhibit proliferation more effectively in *PIK3CA*-mutant breast cancer lines than in wild-type cells [21]. Similarly, the H1047R mutation was a strong predictor of ipatasertib sensitivity across a panel of 100 cell lines [38]. Our observation that ipatasertib is more active in *PTEN*-mutated cells (GBM-002) and lines with high PI3K/AKT pathway aberration burdens is also consistent with existing literature [38, 39]. Furthermore, compensatory signaling through MAPK cascades may explain why sensitivity to PI3K/AKT inhibition varied across the pHGG lines studied [40].

Our population PK modeling successfully characterized the plasma concentration-time profiles of both drugs in mice, revealing a secondary peak in the plasma profile of inavolisib. This presentation is consistent with enterohepatic recirculation, although alternative mechanisms, such as the involvement of multiple absorption transit compartments or drug redistribution from deeper tissue compartments [41], cannot be excluded and may also contribute to this phenomenon.

Our CNS distribution studies were conducted in non-tumor-bearing animals and DIPG xenografts that reproduce the clinical condition of an intact BBB [27, 42]. While both drugs exhibited modest CNS penetration, the high potency of inavolisib, in the low nanomolar range, allowed it to achieve therapeutic brain exposure [43]. A limitation of our CNS distribution studies is that we did not account for plasma protein binding. For inavolisib, protein binding is moderate-to-low in mice (26% unbound) and low in humans (59% unbound) [25]. A recent murine study reported a brain-to-plasma concentration ratio of 0.08 for the free fraction and 0.16 for total drug 1 h after intravenous administration, and these values align with the 0.07 AUC ratio estimated in our study. Generally, drugs with lower plasma protein binding penetrate the CNS more effectively [44].

The relatively low CNS penetration of inavolisib may be partially explained by its status as a substrate for efflux transporters, such as P-glycoprotein and breast cancer resistance protein, at the BBB [25]. While specific CNS distribution data for ipatasertib were previously unavailable, an indirect pharmacodynamic study by Wu et al. demonstrated that ipatasertib inhibits its target, AKT, in brain tissue for at least 24 h [26]. However, that study utilized bioluminescent indicators transfected directly into the striatum via AAVs and did not quantify drug concentrations. It is possible that the concentrations required to inhibit AKT in healthy brain tissue are lower than those needed to suppress malignant cells [26].

To our knowledge, this is the first study to evaluate inavolisib and ipatasertib in primary brain cancers. Ippen et al. reported success using ipatasertib to treat *PIK3CA*-mutant breast cancer brain metastases, significantly increasing the survival of *PIK3CA*-mutant MDA-MB-361 xenografts [45]. However, the tumor microenvironment and vascularity of extracranial metastases differ significantly from primary brain tumors, typically being more “leaky” and thus more permeable to small molecules such as the PI3K/AKT inhibitors [46].

Finally, we previously demonstrated that the heterozygous *H3F3A* K27M mutation can be detected in the ctDNA of CSF in xenografts and cell culture supernatants [27]. The current study expands on this by showing increased ctDNA levels in both media and CSF following inavolisib treatment. This suggests that ctDNA levels in the CSF may rise following successful therapeutic intervention, serving as a potential biomarker for drug response in DMG. This finding warrants further investigation in prospective clinical trials.

## Supplementary Information

Below is the link to the electronic supplementary material.

Supplementary Material 1

## Data Availability

The data that support the findings of this study are available from the corresponding author upon reasonable request.
