# Supplementary material for: CNS distribution and preclinical activity of the PI3K/AKT inhibitors inavolisib and ipatasertib in pediatric-type diffuse high-grade gliomas
Source: Cancer Chemother Pharmacol. 2026 Sep 17;96(1):106. doi: 10.1007/s00280-026-04950-5 (PMC13585860; doi:10.1007/s00280-026-04950-5)
Supplement: Supplementary file 1 [file 280_2026_4950_MOESM1_ESM.docx]

**Supplementary Information**

**CNS distribution and preclinical activity of the PI3K/AKT inhibitors inavolisib and ipatasertib in pediatric-type diffuse high-grade gliomas**

Balaguer-Lluna *et al.*

**Content:**

**Supplementary Method S1.** Quantification of inavolisib and ipatasertib using LC-MS/MS.

**Supplementary Material S1.** Mlxtran code for inavolisib (INAV) oral administration into the enterohepatic recirculation model in plasma, brain and CSF.

**Supplementary Material S2.** Mlxtran code for ipatasertib (IPAT) oral administration in plasma and brain.

**Supplementary Fig. S1.** Angiogenic events in hCMEC/D3 cells exposed to cell culture-conditioned media alone (DIPG-007) or in the presence of inavolisib or ipatasertib at concentrations 1 or 10 µM.

**Supplementary Fig. S2.** Goodness-of-fit diagnostics and visual predictive checks (VPC) for the final plasma, brain, and cerebrospinal fluid (CSF) PK model after oral administration of inavolisib.

**Supplementary Fig. S3.** Goodness-of-fit diagnostics and visual predictive checks (VPC) for the final plasma PK model stratified by the administration route after intravenous and oral administration of ipatasertib.

**Supplementary Fig. S4.** Goodness-of-fit diagnostics and visual predictive checks (VPC) for the final brain PK model following oral administration of ipatasertib.

**Supplementary Fig. S5.** Quantification of human cells in saggital FFPE slides of DIPG-007-bearing brains obtained at the end of treatments.

**Supplementary Fig. S6.** Quantification of cPARP+ cells in the brain of DIPG-007-bearing mouse xenografts treated with 25 mg/kg inavolisib for 14 days.

**Supplementary Table S1.** Patient-derived pHGG models.

**Supplementary Table S2.** Antiproliferative activity of inavolisib and ipatasertib against pHGG lines. IC50 values with 95% confidence intervals (CI).

**Supplementary Table S3.** Comparison of population PK models of inavolisib in plasma during model building.

**Supplementary Table S4.** Population PK parameters of inavolisib in athymic nude mice.

**Supplementary Table S5.** Comparison of population PK models of ipatasertib in plasma and brain during model building.

**Supplementary Table S6.** Population PK parameters of ipatasertib in athymic nude mice.

**Supplementary Methods S1.** Quantification of inavolisib and ipatasertib using LC-MS/MS.

For inavolisib, we used taselisib (Roche) as internal standard and prepared matrix-matched calibration curves in the range 1-5000 ng/mL for plasma and CSF and 3-10,000 ng/g for brain. We precipitated proteins in acetonitrile and injected the supernatants in an Agilent 1290 Infinity II LC system from Agilent Technologies (Santa Clara, CA, USA). We separated the compounds in a C18 column (Zorbax Eclipse Plus 3.0 x 50 mm, 1.8 μm Agilent), kept at 30 ± 0.8 °C. The mobile phase was a mixture of 0.1% formic acid in water (A) and 0.1% formic acid in acetonitrile (B), with the following gradient program: 0 min, 20% B; 4 min, 80% B; 4-5 min, 80% B; 6 min, 20% B; 6-7 min, 20% B (re-equilibration). The flow rate was 300 μL/min. The LC system was coupled to a 6495 triple quadrupole mass spectrometer (LC-QQQ) from Agilent and operated in dynamic multiple reaction monitoring (MRM) mode. The MRM transitions monitored were m/z 408 to m/z 363 for inavolisib and from m/z 461 to m/z 334 for taselisib, which correspond to [M+H]^+^ adducts.

For ipatasertib, we used tolbutamide (Sigma-Aldrich) as internal standard and prepared calibration curves in the range 1-2000 ng/mL for mouse plasma and 2-1000 ng/g for brain. We processed samples by protein precipitation in acetonitrile and injected the supernatants using the autosampler Agilent 1260 Infinity. We separated the compounds in a C18 column (Ascentis Express C18, 2.7 µm, 2.1 x 50 mm, Supelco, Sigma-Aldrich). Mobile phase was a mixture of 0.1% formic acid in water (A) and 0.1% formic acid in acetonitrile (B), with the following gradient program: 0 min, 2% B; 1.2 min, 98% B; 1.2-2 min, 98% B; 2.1 min, 2% B; 2.1-3.5 min, 2% B. Finally, the system was reequilibrated for 2 min. The flow rate was 0.5 mL/min. The detector was a Sciex API 6500 (Sciex, Framingham, MA, USA).

**Supplementary Material S1.**

Mlxtran code for inavolisib (INAV) oral administration into the enterohepatic recirculation model in plasma, brain and CSF.

[LONGITUDINAL]

input = {V,ka, Cl, Q, V2, kbile, kempt, Tfirst, Clbrain, Vbrain, kpb, Clcsf, Vcsf, kpcsf}

PK:

k=Cl/V

k12=Q/V

k21=Q/V2

duration=4

if t>Tfirst & t<Tfirst+duration

kemptying = kempt

else

kemptying = 0

end

compartment(cmt=1, volume=V, concentration=Cc, amount=INAV_p)

absorption(adm=1, cmt=1, ka)

elimination(cmt=1, k)

peripheral(k12, k21)

compartment(cmt=3)

compartment(cmt=4)

transfer(from=1, to=3, kt=kbile)

transfer(from=3, to=4, kt=kemptying)

transfer(from=4, to=1, kt=ka); k_gut=ka

EQUATION:

;parameter definition

;BRAIN

kb0 = Clbrain/Vbrain

CINAV_brain	= INAV_brain/Vbrain

ddt_INAV_brain	= kpb*INAV_p - kb0*INAV_brain

;CSF

kcsf0 = Clcsf/Vcsf

CINAV_csf = INAV_csf/Vcsf

ddt_INAV_csf = kpcsf*INAV_p - kcsf0*INAV_csf

OUTPUT:

output = {Cc,CINAV_brain,CINAV_csf}

**Supplementary Material S2.**

Mlxtran code for ipatasertib (IPAT) oral administration in plasma and brain.

[LONGITUDINAL]

input = {F, Ktr, Mtt, V, Cl, Clbrain, Vbrain, kpb}

EQUATION:

odeType = stiff

; brain concentration

CIPAT_brain = IPAT_brain/Vbrain

; brain elimination rate constant

kb0 = Clbrain/Vbrain

; brain compartment

ddt_IPAT_brain = kpb*IPAT_p - kb0*IPAT_brain

PK:

; central compartment

compartment(cmt = 1,

amount = IPAT_p,

volume = V,

concentration = Cc)

; IV administration

iv(adm = 1, cmt = 1)

; oral administration with transit absorption

oral(adm = 2,

cmt = 1,

Ktr,

Mtt,

p = F)

; plasma elimination

elimination(cmt = 1, Cl)

OUTPUT:

output = {Cc, CIPAT_brain}

**Supplementary Figure S1**. Angiogenic events in hCMEC/D3 cells exposed to cell culture-conditioned media alone (DIPG-007) or in the presence of inavolisib or ipatasertib at concentrations 1 or 10 µM (sub-cytotoxic levels for hCMEC/D3 cells). (A) Representative images of the tube formation assay. From top to bottom, the three lines of images are the bright field, the software-generated diagram of the network (with master junctions in red, branches in green, the mesh in blue, and master segments in yellow), and the immunofluorescence of CD31. (B) Quantification of the total tubule length and number of junctions of the endothelial cell nets. Columns are means and SD of 4 independent assays. Statistics: one way ANOVA with Dunnett's multiple comparisons.

**Supplementary Figure S2.** Goodness-of-fit diagnostics and visual predictive checks (VPC) for the final plasma, brain, and cerebrospinal fluid (CSF) PK model after oral administration of inavolisib. Panels A-C show observed concentrations *versus* population predictions for plasma, brain, and CSF compartments. Panels D-F show observed concentrations *versus* individual predictions. Panels G-I present normalized prediction distribution errors (NPDE) *versus* time after dose. Panels J-L display simulation-based VPCs for plasma, brain, and CSF concentrations. Dots represent observed concentrations. Solid lines correspond to the observed 5^th^, 50^th^, and 95^th^ percentiles, whereas shaded areas represent the corresponding 90% prediction intervals derived from 1000 simulations.

**Supplementary Figure S3**. Goodness-of-fit diagnostics and visual predictive checks (VPC) for the final plasma PK model stratified by the administration route after intravenous and oral administration of ipatasertib. Panels A and B show observed concentrations *versus* population predictions for intravenous (IV) and oral administration, respectively. Panels C and D show observed concentrations *versus* individual predictions. Panels E and F present normalized prediction distribution errors (NPDE) *versus* time after dose. Panels G and H display the VPCs for IV and oral administration, respectively. Dots represent observed concentrations. Solid lines correspond to the observed 5^th^, 50^th^, and 95^th^ percentiles, whereas shaded areas represent the corresponding 90% prediction intervals derived from 1000 simulations.

**Supplementary Figure S4.** Goodness-of-fit diagnostics and visual predictive checks (VPC) for the final brain PK model following oral administration of ipatasertib. Panel A shows observed brain concentrations *versus* population predictions. Panel B shows observed concentrations *versus* individual predictions. Panel C presents normalized prediction distribution errors (NPDE) *versus* time after dose. Panel D displays the simulation-based VPC. D represent observed concentrations. Solid lines correspond to the observed 5^th^, 50^th^, and 95^th^ percentiles, whereas shaded areas represent the corresponding 90% prediction intervals derived from 1000 simulations.

**Supplementary Figure S5**. Quantification of human cells in saggital FFPE slides of DIPG-007-bearing brains obtained at the end of treatments. For inavolisib, terminal samples were obtained after the 14^th^ dose of treatment (day 36). For ipatasertib we obtained brain samples after the 20^th^ dose (day 57). The violin plot graphics are the comparison (Mann-Whitney test) of the mean values of the human cell count obtained for each group, being the dots data from individual mice. Ns: not significant.

**Supplementary Figure S6**. Quantification of cPARP^+^ cells in the brain of DIPG-007-bearing mouse xenografts treated with 25 mg/kg inavolisib for 14 days. (A) Representative images of the brain (mesencephalon) of control and inavolisib-treated animals showing cPARP and anti-human nuclei staining in close sections. Arrow heads indicate cPARP^+^ nuclei. Samples were obtained after the 14^th^ dose of treatment (day 36). (B) Quantification of cPARP^+^ cells. The violin plot graphics are the comparison (Mann-Whitney test) of the cPARP counts for each group, being the dots data from individual mice (5 in each group). Ns: not significant.

**Supplementary Table S1.** Patient-derived pHGG models.

| **Primary culture ID ^a^** | **Sex ^b^** | **Age at diagnosis (years**) | **Original tumor location** | **Original tumor histology** | **pHGG molecular subclass** | **Histone mutation** |
| --- | --- | --- | --- | --- | --- | --- |
| HSJD-DIPG-007 | M | 10 | Pons | pHGG | DMG, H3K27-altered | *H3F3A* K27M |
| HSJD-DIPG-008 | M | 7 | Pons | pHGG | DMG, H3K27-altered | *H3F3A* K27M |
| HSJD-DIPG-011 | F | 3 | Pons | pHGG | DMG, H3K27-altered | *H3F3A* K27M |
| HSJD-DIPG-012 | M | 10 | Pons | pHGG | DMG, H3K27-altered | *H3F3A* K27M |
| HSJD-DIPG-013 | F | 6 | Pons | pHGG | DMG, H3K27-altered | *H3F3A* K27M |
| HSJD-DIPG-014 | F | 8 | Pons | pHGG | DMG, H3K27-altered | *H3F3A* K27M |
| HSJD-DIPG-017 | M | 9 | Pons | pHGG | DMG, H3K27-altered | *H3F3A* K27M |
| HSJD-DIPG-019 | M | 6 | Pons | pHGG | DMG, H3K27-altered | *H3F3A* K27M |
| HSJD-DIPG-021 | F | 6 | Pons | pHGG | DMG, H3K27-altered | *H3F3A* K27M |
| HSJD-DMG-005 | F | 7 | Medulla | pHGG | DMG, H3K27-altered | *H3F3A* K27M |
| HSJD-GBM-001 | F | 11 | Frontal lobe | pHGG | H3-wildtype and IDH-wildtype pHGG | Wild type |
| HSJD-GBM-002 | M | 15 | Hemisphere | pHGG | Diffuse hemispheric glioma H3 G34-mutant | *H3F3A* G34R |

^a^ For clarity purposes, in the manuscript text we omitted the institutional preffix HSJD in the ID of the primary cultures.

^b^ F: female; M: male

**Supplementary Table S2.** Antiproliferative activity of inavolisib and ipatasertib against pHGG lines. IC50 values with 95% confidence intervals (CI).

|  |  | **Inavolisib** |  |  | **Ipatasertib** |  |
| --- | --- | --- | --- | --- | --- | --- |
| **pHGG line** |  | **IC50 (µM)** | **CI** |  | **IC50 (µM)** | **CI** |
| HSJD-DIPG-007 |  | 0.01597 | 0.01305 to 0.01961 |  | 0.8756 | 0.6874 to 1.116 |
| HSJD-DIPG-008 |  | 0.2808 | 0.1288 to 0.6215 |  | 4.399 | 3.188 to 6.014 |
| HSJD-DIPG-011 |  | 0.03901 | 0.02429 to 0.06220 |  | 3.129 | 1.936 to 5.100 |
| HSJD-DIPG-012 |  | 0.3669 | 0.1987 to 0.6939 |  | 9.167 | 6.727 to 12.38 |
| HSJD-DIPG-013 |  | 0.2068 | 0.1263 to 0.3428 |  | 4.543 | 2.950 to 7.170 |
| HSJD-DIPG-014 |  | 0.8483 | 0.5088 to 1.431 |  | 0.5183 | 0.2693 to 0.9485 |
| HSJD-DIPG-017 |  | 0.7713 | 0.5307 to 1.121 |  | 175.3 | 123.2 to 296.4 |
| HSJD-DIPG-019 |  | 0.04691 | 0.03274 to 0.06713 |  | 3.972 | 2.485 to 6.360 |
| HSJD-DIPG-021 |  | 2.017 | 1.192 to 3.404 |  | 7.681 | 4.936 to 11.67 |
| HSJD-DMG-005 |  | 0.1044 | 0.07550 to 0.1444 |  | 13.42 | 9.804 to 18.34 |
| HSJD-GBM-001 |  | 0.996 | 0.8251 to 1.200 |  | 0.6173 | 0.5149 to 0.7407 |
| HSJD-GBM-002 |  | 5.335 | 3.953 to 7.142 |  | 0.5273 | 0.3407 to 0.8235 |

**Table S3.** Comparison of population PK models of inavolisib in plasma during model building.

| **Parameter** | **Model 1** | **Final model** |
| --- | --- | --- |
|  | **TPV (RSE %)** | **TPV (RSE %)** |
| **Fixed effects** |  |  |
| k_a_ (h^-1^) | 1.67 (44.1) | 2.31 (fixed) |
| CL/F (mL/h) | 141.2 (9.5) | 49.8 (15.4) |
| Q/F (mL/h) | - | 125.6 (fixed) |
| V_c_/F (mL) | 67.7 (9.6) | 82.8 (fixed) |
| V_p_/F (mL) | - | 679.3 (fixed) |
| k_bile_ (h^-1^) | 0.5 (20.5) | 0.3 (8.7) |
| k_emptying_ (h^-1^) | 3.6 (87) | 10.1 (16.2) |
| T_first_ (h) | 6.9 (14.3) | 18.1 (11.1) |
| **Random effects** | | |
| IIV_CL/F (%) | 18.2 (91.5) | 35.8 (47.6) |
| IIV_T_first_ (%) | 70.9 (75.0) | 22.9 (44.1) |
| **Residual error** | | |
| RUV_plasma (ng/mL) | 0.4 (24.6) | 0.36 (26.3) |
| **Model discrimination criteria** | | |
| OFV (-2LL) | 489.6 | 395.1 |
| AIC | 495.6 | 407.1 |
| BIC | 499.8 | 414.2 |

**Abbreviations**. AIC: Akaike information criteria; BIC: Bayesian information criteria; CL/F: apparent clearance of inavolisib from plasma; IIV: interindividual variability approximated to the coefficient of variation (%), calculated using the expression $\sqrt{e^{\omega^{2}}-1}*100$, where ω^2^ is the estimated variance; ka: absorption rate constant; k_bile_: transfer rate constant between the central compartment and the bile; k_emptying_: transfer rate constant between the bile and the gut; OFV: objective function value; Q/F: apparent intercompartmental clearance between central and peripheral compartment; RSE: relative standard error; RUV_plasma: residual unexplained variability of inavolisib in plasma; TPV: typical population value; V_c_/F, V_p_/F: apparent volume of distribution of inavolisib in the central and peripheral compartment, respectively.

**Supplementary Table S4.** Population PK parameters of inavolisib in athymic nude mice.

| **Pharmacokinetic parameter** | **Population value** | **RSE %** | **Model Building Step** |
| --- | --- | --- | --- |
| k_a_ (h^-1^) | 2.31 | Fixed | 1 |
| CL/F (mL/h) | 49.8 | 15.4 | 1 |
| Q/F (mL/h) | 125.6 | Fixed | 1 |
| V_c_/F (mL) | 82.8 | Fixed | 1 |
| V_p_/F (mL) | 679.3 | Fixed | 1 |
| k_bile_ (h^-1^) | 0.3 | 8.7 | 1 |
| k_emptying_ (h^-1^) | 10.1 | 16.2 | 1 |
| T_first_ (h) | 18.1 | 11.1 | 1 |
| CL_brain_ (mL/h) | 12.0 | 38.0 | 2 |
| V_brain_ (mL) | 3.2 | 25.6 | 2 |
| k_pb_ (h^-1^) | 0.01 | 35.1 | 2 |
| CL_CSF_ (mL/h) | 9.1 | 21.5 | 3 |
| V_CSF_ (mL) | 0.036 | fixed | 3 |
| k_pCSF_ (h^-1^) | 2 | fixed | 3 |
| **Random effects** |  | | |
| IIV_CL (%) | 35.8 | 47.6 | 1 |
| IIV_T_first_ (%) | 22.9 | 66.8 | 1 |
| IIV_CL_brain_ (%) | 45.1 | 23.8 | 2 |
| IIV_CL_CSF_ (%) | 51.6 | 28.7 | 3 |
| RUV_plasma (ng/mL) | 0.36 | 26.3 | 1 |
| RUV_brain (%) | 24.0 | 40.6 | 2 |
| RUV_CSF (ng/mL) | 11.7 | fixed | 3 |

**Abbreviations**. CL/F: apparent clearance of inavolisib from plasma; CL_brain_: clearance of inavolisib fom the brain; CL_CSF_: clearance of inavolisib from the CSF; IIV: inter-individual variability approximated to the coefficient of variation (%) calculated using the expression $\sqrt{e^{\omega^{2}}-1}*100$, where ω^2^ is the estimated variance; k_a_: absorption rate constant; k_bile_: transfer rate constant between the central compartment and the bile; k_pb_: rate of inavolisib transfer from plasma to the brain; k_emptying_: transfer rate constant between the bile and the gut; k_pCSF_: rate of inavolisib transfer from plasma to the CSF; Q/F: apparent intercompartmental clearance between central and peripheral compartment; RSE%: % relative standard error; RUV_brain, RUV_CSF, RUV_plasma: residual unexplained variability of inavolisib in brain, CSF and plasma; T_first_: time corresponding to the secondary peak concentration in the concentration-time profile; V_c_/F, V_p_/F: apparent volume of distribution of inavolisib in the central and peripheral compartment, respectively; V_brain_, V_CSF_: volume of distribution of inavolisib in the brain and CSF.

**Supplementary Table S5**. Comparison of population PK models of ipatasertib in plasma and brain during model building.

| **Parameter** | **Model 1** | **Final model** |
| --- | --- | --- |
|  | **TPV (RSE %)** | **TPV (RSE %)** |
| **Fixed effects** |  |  |
| F | 0.56 (59.6) | 0.23 (19.2) |
| k_a_ (h⁻¹) | 0.094 (51.6) | - |
| K_tr_ (h⁻¹) | - | 0.04 (40.3) |
| MTT (h) | - | 4.51 (37.6) |
| V (mL) | 57.5 (20.1) | 95.0 (21.0) |
| CL (mL/h) | 27.1 (14.8) | 17.7 (24.0) |
| CL_brain_ (mL/h) | 0.56 (44.2) | 7.12 (32.0) |
| V_brain_ (mL) | 0.72 (88.1) | 3.73 (34.0) |
| K_pb_ (mL/h) | 0.003 (34) | 0.016 (22.1) |
| **Random effects** | | |
| IIV_F (%) | 57.9 (19.8) | 40.5 (27.3) |
| IIV_k_a_ (%) | 115 (22.1) | - |
| IIV_K_tr_ (%) | - | 131.5 (30.8) |
| IIV_MTT (%) | - | 108.4 (22.0) |
| IIV_V (%) | 58.8 (74.6) | 60.3 (31.1) |
| IIV_CL (%) | 57 (27.9) | 71.1 (22.5) |
| IIV_CL_brain_ | 30.2 (98.0) | 82.5 (20.5) |
| IIV_k_pb_ | 49.0 (32) | 60.5 (21.5) |
| **Residual error** | | |
| RUV_plasma (%) | 36.9 (14.6) | 37.0 (16.7) |
| RUV_brain (%) | 33 (44.1) | 23.1 (38.9) |
| **Model discrimination criteria** | | |
| OFV (-2LL) | 1183.0 | 1151.5 |
| AIC | 1201.0 | 1173.5 |
| BIC | 1219.1 | 1195.6 |

**Abbreviations**. AIC: Akaike Information Criterion; BIC: Bayesian Information Criterion; CL: clearance; CL_brain_: clearance of ipatasertib from the brain; F: oral bioavailability; IIV: interindividual variability approximated to the coefficient of variation (%), calculated using the expression $\sqrt{e^{\omega^{2}}-1}*100$, where ω^2^ is the estimated variance; k_a_: first-order absorption rate constant; K_tr_: transit rate constant; MTT: mean transit time; OFV: objective function value; RSE: relative standard error; RUV_brain, RUV_plasma: residual unexplained variability of ipatasertib in brain and plasma; TPV: typical population value; V_brain_, V: volume of distribution of ipatasertib in the brain and central compartment.

**Supplementary Table S6.** Population PK parameters of ipatasertib in athymic nude mice.

| **Pharmacokinetic parameter** | **Population value** | **RSE %** | **Model Building Step** |
| --- | --- | --- | --- |
| F | 0.23 | 19.2 | 1 |
| K_tr_ (h^-1^) | 0.04 | 40.3 | 1 |
| MTT (h) | 4.51 | 37.6 | 1 |
| V (mL) | 95.04 | 21.0 | 1 |
| CL (mL/h) | 17.65 | 24.0 | 1 |
| CL_brain_ (mL/h) | 7.12 | 32.0 | 2 |
| V_brain_ (mL) | 3.73 | 34.0 | 2 |
| k_pb_ (mL/h) | 0.016 | 22.1 | 2 |
| **Random effects** |  | | |
| IIV_F (%) | 40.5 | 27.3 | 1 |
| IIV _K_tr_ (%) | 131.5 | 30.8 | 1 |
| IIV _MTT (%) | 108.4 | 22.0 | 1 |
| IIV _V (%) | 60.3 | 31.1 | 1 |
| IIV _CL (%) | 71.1 | 22.5 | 1 |
| IIV _CL_brain_ (%) | 82.5 | 20.5 | 2 |
| IIV _k_pb_ (%) | 60.5 | 21.5 | 2 |
| RUV_ plasma (%) | 37.0 | 16.7 | 1 |
| RUV_brain (%) | 23.1 | 38.9 | 2 |

Abbreviations: CL, CL_brain_: clearance of ipatasertib from plasma and from the brain; F: oral bioavailability; IIV: interindividual variability approximated to the coefficient of variation (%), calculated using the expression $\sqrt{e^{\omega^{2}}-1}*100$, where ω^2^ is the estimated variance; k_pb_: plasma-to-brain transfer rate constant; K_tr_: transit rate constant; MTT: mean transit time compartment; RSE%: relative standard error; RUV_brain, RUV_plasma: residual unexplained variability of ipatasertib in brain and plasma; V_brain_, V: volume of distribution of ipatasertib in the brain and central compartment.
